# *Cluster-mining*: an approach for determining core structures of metallic nanoparticles from atomic pair distribution function data

**DOI:** 10.1107/S2053273319013214

**Published:** 2020-01-01

**Authors:** Soham Banerjee, Chia-Hao Liu, Kirsten M. Ø. Jensen, Pavol Juhás, Jennifer D. Lee, Marcus Tofanelli, Christopher J. Ackerson, Christopher B. Murray, Simon J. L. Billinge

**Affiliations:** a Department of Applied Physics and Applied Mathematics, Columbia University, New York, NY 10027, USA; b Department of Chemistry, University of Copenhagen, Copenhagen, DK-2100, Denmark; c Computational Science Initiative, Brookhaven National Laboratory, Upton, NY 11973, USA; d Department of Chemistry, University of Pennsylvania, Philadelphia, PA 19104, USA; e Department of Chemistry, Colorado State University, Fort Collins, CO 80523, USA; f Department of Materials Science and Engineering, University of Pennsylvania, Philadelphia, PA 19104, USA; g Condensed Matter Physics and Materials Science Department, Brookhaven National Laboratory, Upton, NY 11973, USA

**Keywords:** structural models, nanoparticles, clusters, pair distribution functions, data mining, screening

## Abstract

A novel approach for finding and evaluating structural models of small metallic nanoparticles is presented. Rather than fitting a single model with many degrees of freedom, libraries of clusters from multiple structural motifs are built algorithmically and individually refined against experimental pair distribution functions. Each cluster fit is highly constrained. The approach, called *cluster-mining*, returns all candidate structure models that are consistent with the data as measured by a goodness of fit. It is highly automated, easy to use, and yields models that are more physically realistic and result in better agreement to the data than models based on cubic close-packed crystallographic cores, often reported in the literature for metallic nanoparticles.

## Abstract 2

A novel approach for finding and evaluating structural models of small metallic nanoparticles is presented.

## 1. Introduction   

Advances in the synthesis of metallic nanoparticles have given researchers a great deal of control in tailoring their functionalities for many applications including catalysis (Lewis, 1993 ▸; Somorjai & Park, 2008 ▸), plasmonics (Atwater & Polman, 2010 ▸; Linic *et al.*, 2011 ▸), energy conversion (Aricò *et al.*, 2005 ▸) and biomedicine (Rosi & Mirkin, 2005 ▸; Ackerson *et al.*, 2006 ▸; Nune *et al.*, 2009 ▸). At the simplest level, the distinct properties of nanoparticles can be attributed to the increased role of their external surfaces, which can be manipulated by changing experimental parameters in a synthesis to obtain particles of a certain size, shape and composition. However, an atomic scale characterization of the varying structural degrees of freedom, including size, morphology and chemical ordering of very small nanoparticles, remains a major challenge (Bøjesen & Iversen, 2016 ▸; Lee *et al.*, 2016 ▸). Critical to engineering the next generation of these materials by design, rather than empirical optimization, is to develop structural probes and modeling methodologies capable of quantifying the arrangements of atoms at the smallest length scales possible.

Determining the atomic core structures of ultra-small nanoparticles using X-ray powder diffraction methods is difficult (Billinge & Levin, 2007 ▸). The information obtained in these experiments is degraded not only because of finite size effects but also because the internal arrangements of atoms deviate significantly from bulk materials. Non-crystallographic structures have long been reported in electron microscopic studies of metallic nanoparticles (Ino, 1966 ▸; 1969 ▸; Marks & Howie, 1979 ▸; Sun & Xia, 2002 ▸; Chen *et al.*, 2013 ▸) and it is established that growth mechanisms across a diversity of synthesis methods are directed by the size-dependent formation and rearrangement of multiply twinned domains, in addition to thermodynamic stabilization of nanoparticle surfaces by capping agents (Lofton & Sigmund, 2005 ▸; Langille *et al.*, 2012 ▸; Marks & Peng, 2016 ▸). Despite this evidence, atomic models built from face-centered cubic (f.c.c.) cores, which do not account for the multi-domain nature of these materials, are still commonly used in atomic pair distribution function (PDF) analysis of metallic nanostructures (Petkov & Shastri, 2010 ▸; Page *et al.*, 2011 ▸; Kumara *et al.*, 2014 ▸; Fleury *et al.*, 2015 ▸; Wu *et al.*, 2015 ▸; Poulain *et al.*, 2016 ▸; Petkov *et al.*, 2018 ▸).

It was recently demonstrated that the PDF does contain information allowing for the detection and characterization of internal atomic interfaces in a diversity of metallic nano­materials and atomic clusters (Banerjee *et al.*, 2018 ▸). It was also shown that the PDF could differentiate between various arrangements of multiply twinned domains. For a majority of the samples surveyed, simple decahedral or icosahedral cluster cores, instead of f.c.c. attenuated crystal (AC) approximations or single-crystal f.c.c. cutouts, gave significantly improved fits. This analysis hinged on time-consuming, manual trial-and-error refinements of a few representative cluster models from different structure motifs. Here we describe a new approach for determining the best models for metallic nanoparticle core structures by automatically generating large numbers of candidate cluster structures and comparing them with PDF data from nanoparticles. The methodology differs from traditional approaches for crystallographic analysis of nanoparticles where a single model containing many refinable parameters is used to fit peak profiles from a measured diffraction pattern. Instead, this approach uses many structure models and highly constrained refinements to screen libraries of discrete clusters against experimental PDF data, with the aim of finding the most representative cluster structures for the ensemble average nanoparticle from any given synthesis.

## 2. Modeling   

The core of the new approach is to generate large numbers of candidate structure models, which in principle could be pulled from databases or generated algorithmically. PDFs are then computed from each model and compared with a measured PDF. A small number of refinable parameters may be varied in this last comparison step, such as an overall scale factor and an average bond length, in such a way as to minimize an agreement factor, *R*_w_, described in greater detail below. The results of the comparisons for all models are then reported back to the experimenter. In this initial implementation we tested finite-sized cluster models, which we use to compare against data collected from small metallic nanoparticle samples, and in this case we generate the libraries of clusters, which we call cluster mines, algorithmically.

Clusters may be grouped into different types, or motifs, which have specific algorithmic structure builders. Here we consider motifs built from densely packed hard-sphere models which form a seed or atomic core for the metallic nanoparticles of interest.

Three dense-packing configurations were used in this study (*N* specifies the smallest building block for the atomic core): (1) the cubic close-packed (c.c.p.) tetrahedron (*N* = 4) yielding f.c.c. clusters (Kepler, 1611 ▸; Hales, 2005 ▸), (2) the pentagonal bipyramid (*N* = 7), which generates decahedral clusters (Bagley, 1965 ▸), and (3) the icosahedron (*N* = 13) used to build magic or Mackay icosahedra (Mackay, 1962 ▸).

A diversity of different cluster geometries can be made by stacking layers of atoms in specific arrangements on top of the densely packed atomic seeds and by truncating the growth along different high-symmetry directions (Martin, 1996 ▸). These structure-building algorithms are implemented in the *Atomic Simulation Environment* (*ASE*) Python package (Hjorth Larsen *et al.*, 2017 ▸), and other motifs are currently being developed. A fourth motif, singly twinned f.c.c. bicrystals, was also built and tested by applying a simple transformation to f.c.c. single-crystal clusters. Briefly, f.c.c. clusters are cut along a {111} lattice plane and misoriented by applying a 60° rotation to one half of the crystal around an axis normal to the {111} plane. This is carried out on f.c.c. crystals with an odd number of c.c.p. layers such that one {111} contact twin plane, resulting in two mirror-equivalent domains with the same number of atoms, is generated. In this way, popular cluster types from the literature are created and added to the mine, but this also illustrates how other cluster types may be generated and added in the future.

The geometries that result from the different motif-specific truncation criteria can be classified as families, which share the same local atomic environment common to each motif but differ in the topology of their polyhedral surfaces. For example, in the *ASE* decahedron structure builder, four parameters can uniquely specify a cluster model: a nearest-neighbor bond distance, the number of layers parallel to the fivefold axis, the number of layers truncated perpendicular to the five pentagonal edges and the number of layers truncated perpendicular to the five apical vertices. When no truncation exists, regular decahedra or pentagonal bipyramids are generated, whereas truncation of the pentagonal edges produces families of Ino-truncated wire-like decahedra (Ino, 1966 ▸) and apical truncation yields Marks decahedra (Marks, 1994 ▸) with re-entrant facets. Changing the type and degree of truncation influences the resulting morphology of the cluster, and in decahedra this also changes the relative number of atoms within the five f.c.c.-like subunits *versus* the atoms situated at twin boundaries between the decahedral domains and at surfaces.

If a unique set of parameters that specify a cluster model is given as input to a structure builder in *ASE*, a list of Cartesian coordinates is returned which may be read into a PDF calculating program. In this case we use our own complex modeling infrastructure, *CMI* (Juhás *et al.*, 2015 ▸). PDFs are then calculated from the atomic coordinates using the Debye scattering equation (DSE; Debye, 1915 ▸) PDF calculator implemented in *DiffPy*’s DebyePDFCalculator class under *SrFit*. The atomic coordinates in space are held constant in the refinements but four parameters are allowed to vary to obtain good agreement between the calculated and measured PDFs: an isotropic expansion coefficient (linear scaling in *r*) to account for differences in nearest-neighbor distances, a single *U*_iso_ (isotropic atomic displacement parameter), a single scale factor and a parameter for correlated motion effects, δ_2_ (Proffen & Billinge, 1999 ▸). Parameters that describe the resolution of the measurement (*Q*_damp_ and *Q*_broad_) are obtained by independently refining a bulk calibrant measured in the same geometry as the nanocrystalline sample and fixed.

The cluster mine is built by iterating through the integer values for parameters and combinations thereof, specifying the number of added and truncated layers for each motif-specific structure builder. The size of the structure mine (the number of clusters in the mine) can be tuned by providing bounds on the values that a given builder parameter may take or by specifying a minimum and maximum number of atoms (*N*_a_) in the clusters regardless of the builder. During this procedure, *cluster-mining* stores metadata such as the number of atoms, atom type, nearest-neighbor distance and motif, and starting values for the refinable variables along with the set of integers for that cluster. This information is then passed to *ASE* which generates the *x*, *y*, *z* atomic coordinates, which are then used as inputs to *CMI* to calculate the PDF and refine the variable parameters against a measured PDF for each cluster in the mine. The fit range in *r* can also be adjusted prior to refining the library of clusters. The *cluster-mining* program then returns a table of initial and refined PDF parameter values, and goodness of fit (*R*_w_), with each individual refinement linked to the input cluster parameters and associated metadata. A plot can then be generated of the best fit *R*_w_ *versus* the number of atoms (*N*_a_) for all clusters in the mine. We call this plot the cluster-screen map. The cluster-screen map can be filtered or labeled according to any cluster-specific metadata, such as the motif.

The dimension of the input parameter space (typically 3–6) is significant, so the size of the mine can be large. For example, 2419 unique combinations are possible for decahedra containing less than 1500 atoms, including regular, Ino, Marks and Ino-truncated Marks families. However, the *cluster-mining* method is easily parallelizable and lends itself to deployment on multi-node computers. As well as giving more ideal cluster model fits than, for example, stochastic approaches (Page *et al.*, 2011 ▸), the procedure greatly speeds up a researcher’s workflow compared with more manual trial-and-error routines. This approach to nanostructure modeling may also be sped up by increasing the efficiency of selection of the clusters from the mine for testing and we expect that statistical approaches such as machine learning will be effective in this regard, though this is beyond the scope of this article.

## 3. Results   

**Figure 1:**
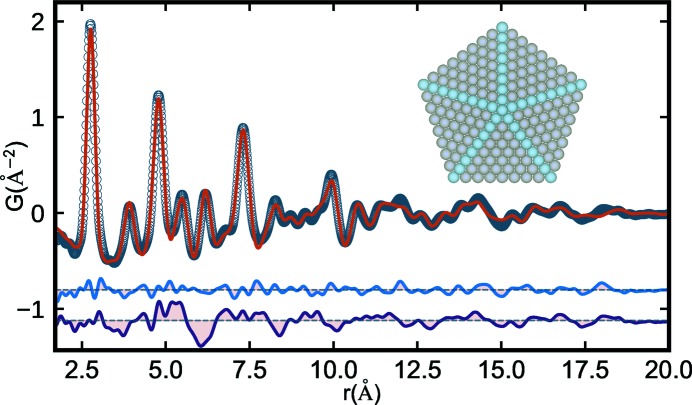
Experimental PDF (open circles) from ∼3 nm Pd nanoparticles and the calculated PDF (red solid line) from a 3.6 nm decahedron (inset). Offset below are the difference curves from the discrete decahedral (blue) and spherically attenuated f.c.c. crystal model (dark purple) refined to the measured Pd nanoparticle data.

We first applied our *cluster-mining* approach to a PDF measured from ∼3 nm Pd nanoparticles that was described by Banerjee *et al.* (2018 ▸). In that work, the best cluster model that was found was a 609-atom regular decahedron with a maximum inter-vertex distance of 36.4 Å. This was determined by trial-and-error testing of a regular decahedral size series, starting with a 22.8 Å (181-atom) decahedron and ending with an 51.9 Å (1442-atom) decahedron. The refinement of the best-fit decahedral cluster core for the small Pd nanoparticles is given in Fig. 1 ▸, which shows the experimental nanoparticle PDF and the calculated PDF for the 609-atom decahedron, with the cluster structure reproduced in the inset. The difference curves (fit residuals) for both the discrete cluster and f.c.c. AC (attenuated crystal) models are offset below in blue and dark purple, respectively.

**Figure 2:**
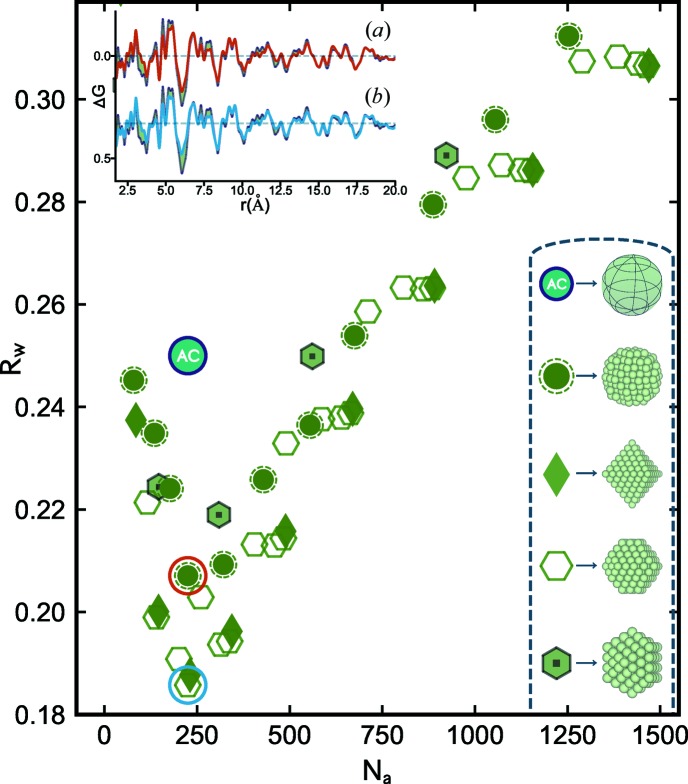
Scatter plot of agreement factors (*R*_w_) for discrete f.c.c. clusters fitted to the Pd nanoparticle PDF, plotted as a function of the number of atoms per model (*N*_a_). Each point is an individual PDF refinement of a discrete structure from a different f.c.c. cluster type. These have been categorized as different families (see Section 2 for details) which are represented in the legend at the bottom right. From top to bottom, the five families from the f.c.c. motif shown here are AC, discrete spheres, regular octahedral, truncated octahedral and cuboctahedral. In the scatter plot, the AC model fit is marked as a solid teal circle, and the best-fit model from the discrete spherical and truncated octahedral families is highlighted with red and blue circles, respectively. In the inset to the top left, the PDF fit residual from the AC model (light purple) is overlaid with the difference curves from the aforementioned best-fit discrete sphere (*a*) and octahedral clusters (*b*), using the same colors as highlighted in the scatter plot.

In the work by Banerjee *et al.* (2018 ▸), it was demonstrated that a diversity of small, representative clusters from motifs with different domain structures and morphologies were needed to fit all the metallic nanoparticle PDFs that were considered. However, it is a laborious task to find the best cluster models and it would also be valuable to know about the degeneracy of the solution set *i.e.* how many different clusters give comparable agreement with the data. To do this we can construct libraries, or mines, containing hundreds to thousands of discrete cluster models. These were built combinatorially from motif-specific structure builders as described in Section 2. To demonstrate what can be learned from this approach we applied it to the measured PDF from Pd nanoparticles shown in Fig. 1 ▸ by generating and fitting 464 different discrete models. We start by investigating 60 clusters from a single structure motif (f.c.c.) in greater detail. The results are summarized in Fig. 2 ▸, which shows the best-fit agreement factor of each f.c.c. model plotted *versus* the number of atoms in the model (*N*_a_), which we call a cluster-screen map. We compare the cluster-mined solutions to that from the f.c.c. AC model, which is the benchmark for refinements carried out in the traditional way using *PDFgui*. For this Pd nanoparticle sample, the AC model resulted in an *R*_w_ of 0.253 and this value is shown as a solid teal circle in Fig. 2 ▸. This fit was obtained with a refined spherical particle diameter of 19.4 Å, which corresponds to *N*_a_ ≃ 225 for a discrete f.c.c. spherical cutout. Next we built discrete spherical f.c.c. cutouts to compare with the AC model. These are shown as solid green circles with a dashed outline in Fig. 2 ▸. This family of clusters has *R*_w_’s that follow a trend with nanoparticle size. The trend goes through a minimum at a particle size containing *N*_a_ = 225, the same as the AC model.

Somewhat surprisingly, the *R*_w_ of this model was lower than that of the AC model, though both correspond to spheres of f.c.c. material. There are a number of differences between calculating the PDF of a spherical particle using a discrete spherical cluster and the DSE *versus* a bulk model attenuated with the characteristic function of a sphere. One of the largest factors to affect the *R*_w_ appears to be the choice of *Q*_min_ used in the DSE calculation. This strongly influences the baseline in the PDF (Farrow & Billinge, 2009 ▸) depending on the degree to which the small-angle scattering signal is incorporated into the measured and calculated PDFs. Understanding this effect in detail is beyond the scope of this article, but tests on this Pd nanoparticle sample show that the best *R*_w_ factors were obtained when the same *Q*_min_ was used for the DSE calculations as was used in the treatment of the measured data. We note that this careful study of spherical nanoparticle models yields insight into how the different cluster models work with the data, and improvements in fit are possible over the AC model. However, as was pointed out by Banerjee *et al.* (2018 ▸), the spherical models do not remove much of the signal from residuals and are still deficient in many regards.

We now turn to models with the same f.c.c. atomic structure, but which are cut out from the bulk with well defined surface faceting. The clusters considered here were made by forming octahedral shapes exhibiting {001} and {111} facets. Three families of faceted f.c.c. octahedra are shown in Fig. 2 ▸: regular octahedra (solid diamonds) with only {111} facets exposed, truncated octahedra (hexagons) with a mixture of {111} and {001} surfaces, and cuboctahedra (solid hexagons) which satisfy a specific truncation condition where the percentage of the surface covered by {001} (non-close-packed) facets is largest and all facet edges contain the same number of atoms. The cuboctahedral family of clusters has the most isotropic or spherical shape from the octahedral motif. There are subtle variations in the *R*_w_ trends for each of the faceted f.c.c. octahedral families, with the cuboctahedral series following most closely the results of the discrete f.c.c. spheres. Regular and truncated octahedra follow trends that are offset slightly below the spherical and cuboctahedral series. Overall, the f.c.c. cluster families track very closely with each other, reaching *R*_w_ minima in the vicinity of *N*_a_ ≃ 250 and in fact the best candidate faceted octahedron is a slightly truncated cluster with 225 atoms, which has the same *N*_a_ as the best-fit discrete f.c.c. sphere and AC approximation. In the inset of Fig. 2 ▸ we compare the fit residuals between the f.c.c. AC model and (*a*) the minimum *R*_w_ f.c.c. sphere and (*b*) the faceted octahedron, respectively. Although improvements are seen in *R*_w_, it is clear that the majority of the misfit signal in the residual is not affected. This suggests that collectively, monocrystalline f.c.c. cluster cores regardless of shape might not be the most suitable structure motif for the small Pd nanoparticles studied here.

**Figure 3:**
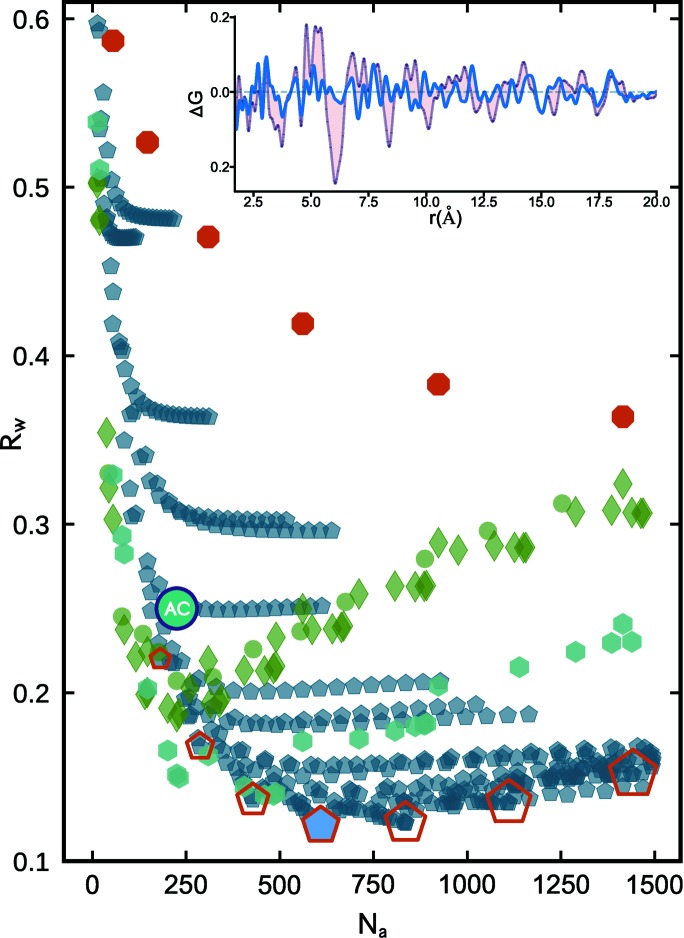
Scatter plot of agreement factors (*R*_w_) for discrete clusters from three different structure motifs fitted to the Pd nanoparticle PDF, plotted as a function of the number of atoms per model (*N*_a_). Green diamonds and circles are for the f.c.c. motif and include the faceted and spherical cluster families shown in Fig. 2 ▸. Red octagons are for Mackay icosahedra, teal hexagons are for singly twinned f.c.c. bicrystals and blue pentagons are for different decahedral families (see text for details). The best-fit AC model is marked as a solid blue circle. Red pentagons outline a size series of regular decahedra (pentagonal bipyramids). In the inset, the PDF fit residual from the AC model (light purple) is overlaid with the difference curve from the absolute best-fit cluster model, which in this case is the 609-atom non-truncated decahedron (Fig. 1 ▸ inset).

Next, twinned cluster models from decahedral, icosahedral and singly twinned structure motifs were constructed and added to the mine, and compared with the Pd nanoparticle data. In Fig. 3 ▸ we reproduce the same *R*_w_ scatter plot as discussed for f.c.c. cutouts in Fig. 2 ▸ with each point appearing as green symbols.

The blue symbols are from 398 different decahedral structures including regular decahedra (pentagonal bipyramids), Ino decahedra, Marks decahedra and Ino-truncated Marks decahedra (see Section 4 for additional details). The red symbols are from icosahedral structures and the teal symbols (hexagons) are from singly twinned f.c.c. bicrystals. 55% of the decahedral models tested are in better agreement with the measured Pd nanoparticle PDF than the best-fit faceted f.c.c. octahedron. This can be seen as many of the blue symbols are at lower *R*_w_ values than the lowest green symbol in the cluster-screen map.

The best candidate decahedral models for the Pd nanoparticle data turn out to be from a family of pentagonal bipyramids. The *R*_w_ points from this family are outlined with red pentagons in Fig. 3 ▸. These clusters increase in diameter, or maximum intervertex distance, as a function of *N*_a_ and reach a minimum *R*_w_ of 0.121 for a decahedron with 609 atoms and a diameter of ∼3.6 nm, which is nearly twice the size of the best f.c.c. model and contains 384 more atoms. This diameter for the 609-atom decahedron is much closer to the transmission electron microscopy (TEM) estimated particle size of 3.0 ± 0.3 nm for the Pd nanoparticles investigated here. The TEM estimate is not a full sample average and is a slight underestimate of the average particle size. This may be because the TEM estimate is averaging over particles viewed from different directions, and the particles are somewhat oblate in shape. The shape of this 609-atom decahedron (Fig. 1 ▸ inset) also aligns with the observation of oblate-like morphologies in high-resolution TEM images of these Pd nanoparticles (Banerjee *et al.*, 2018 ▸). In general, combined imaging and sample average estimates of particles are preferable for building a full picture. Most convincingly, in comparing the fit residual from the f.c.c. AC model and the best-fit decahedron (Fig. 3 ▸ inset) we observe drastic changes to the largest amplitude features in the difference curve, with many of the misfit correlations removed altogether, which strongly supports the idea that the decahedral cluster core is capturing the correct modification to the f.c.c. structure. The ability to determine nanoparticle structure and morphology in such detail can be expected to yield insights into questions such as the mechanisms governing nanoparticle formation and stability (Ringe *et al.*, 2013 ▸) through systematic studies of well controlled nanoparticle systems under different growth conditions.

It is often discussed in the literature whether the range of *r* where features are seen in the PDF corresponds to a range of structural coherence or a crystallite size but this modeling shows how such a situation may come about. The observed PDF structural coherence range is roughly the size of one of the five f.c.c. sub-domains that make up the decahedral cluster. This is an exemplar case where a model of a much larger cluster, which accounts for the inter-domain structure and domain twin boundaries, produces a significantly better fit to the PDF than just a model of incoherent small grains of f.c.c. material and provides an illustration of how rather small nanoclusters may consist of sub-domains in general. The other cyclic twinned motif tested in Fig. 3 ▸, magic icosahedra (red markers), yields *R*_w_’s that are significantly worse than both the f.c.c. and decahedral motifs, which shows that despite containing a high density of contact twin boundaries, the spatial arrangement of these domains is not representative for this Pd nanoparticle sample and the icosahedral motif can be easily ruled out. Singly twinned f.c.c. bicrystals follow a trend that is intermediate between the single-crystal f.c.c. cutouts and the best candidate decahedral models, which makes sense given that the density of atoms on twin planes is also intermediate between the two.

**Figure 4:**
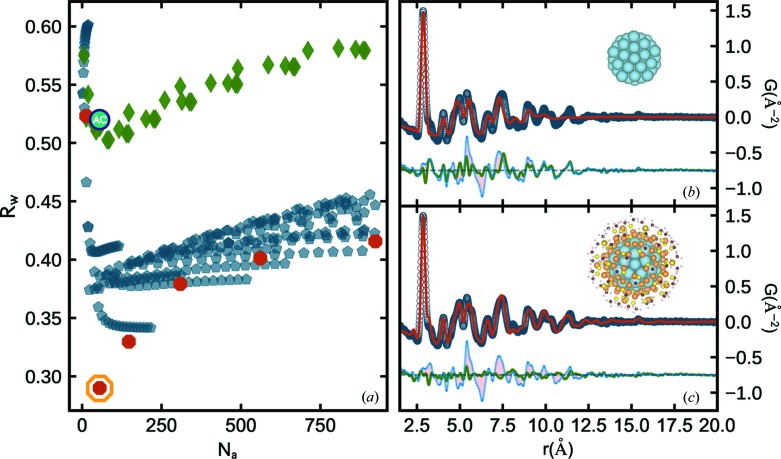
(*a*) Cluster-screen map for Au_144_(SC6)_60_ including structures from AC (teal), f.c.c. octahedral (green), decahedral (blue) and icosahedral (red) motifs. The best-fit cluster core, a 55-atom Mackay icosahedron, is outlined in orange. (*b*) Measured PDF (open circles) from the Au_144_(SC6)_60_ cluster sample and the calculated PDF (red solid line) from the cluster-mined 55-atom Mackay core (shown in inset). The difference curve from this refinement is offset below in green and overlaid with the AC residual in light blue. (*c*) Analogous to (*b*), except the calculated PDF (red solid line) is from a DFT-derived structure solution (Lopez-Acevedo *et al.*, 2009 ▸) for Au_144_(SC6)_60_, which shares the icosahedral core shown in (*a*), and also contains lower-symmetry outer layers. In the inset, the radii of atoms surrounding the DFT-determined core are scaled down by a factor of two for illustration purposes.

We now apply *cluster-mining* to a series of ultra-stable magic sized Au_144_(SR)_60_ clusters (Whetten *et al.*, 1996 ▸) prepared with different thiolate ligands (Ackerson *et al.*, 2010 ▸; Qian & Jin, 2011 ▸). In Fig. 4 ▸(*a*) we show the cluster-screen map from one sample in this series consisting of hexanethiol-ligated clusters, Au_144_(SC6)_60_. In this case, icosahedral structures perform better than the AC, f.c.c. octahedral and decahedral motifs. The best-fit model obtained is a 55-atom Mackay icosahedron with *R*_w_ = 0.228, highlighted with an orange outline in the cluster-screen map, Fig. 4 ▸(*a*). In Fig. 4 ▸(*b*) we show the PDF of the best-fit cluster-mined 55-atom core. The difference curve is offset below and overlaid on the difference curve from the f.c.c. AC approximation. The main misfit in the AC difference curve between 5 and 8 Å is drastically improved and no other clusters are close in agreement, giving us confidence that the core of this Au_144_ cluster is icosahedral in nature.

In this case, a structure solution for Au_144_(SC6)_60_ has been found by density functional theory (DFT), high-angle annular dark-field scanning transmission electron microscopy (HAADF-STEM) and PDF analysis (Lopez-Acevedo *et al.*, 2009 ▸; Bahena *et al.*, 2013 ▸; Jensen *et al.*, 2016 ▸). In Fig. 4 ▸(*c*) we show the PDF from the 144-atom Lopez-Acevedo (LA) model, which contains chiral arrangements of atoms on top of a core that is nearly identical to a Mackay icosahedron (Jensen *et al.*, 2016 ▸; Banerjee *et al.*, 2018 ▸). The additional lower-symmetry outer layers of the LA model further remedy the misfit features at higher *r* [Fig. 4 ▸(*c*)] and improve the overall agreement factor to a value of *R*_w_ = 0.146. This highlights the fact that *cluster-mining* can also identify good candidate cluster cores, which can be used as starting structures for more complex core/shell models.

**Figure 5:**
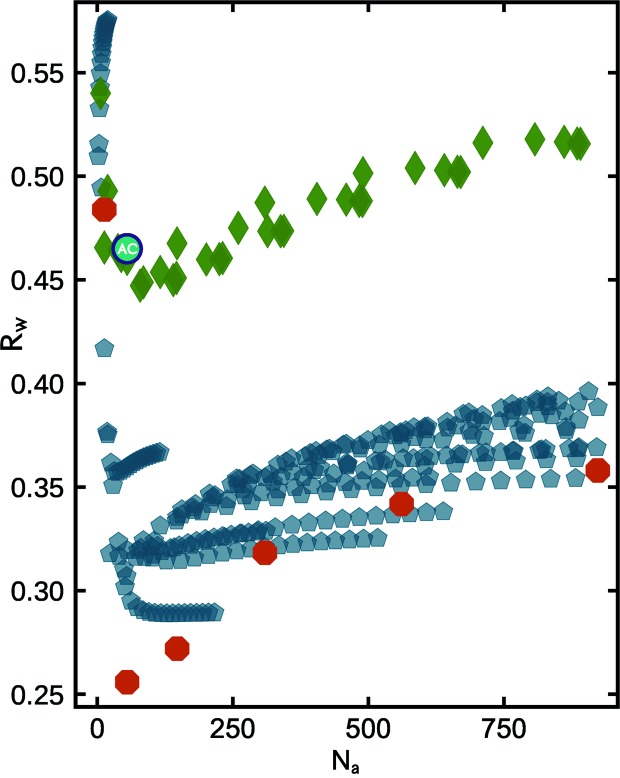
Cluster-screen map for a multi-phase cluster sample, Au_144_(SC6)_60_. The cluster mine includes AC (teal), f.c.c. octahedral (green), decahedral (blue) and icosahedral (red) motifs.

Not all samples are ideally single phase and we would like to know how robust the *cluster-mining* approach is in the case where more than one phase exists in the sample. This can be tested using an Au_144_(SR)_60_ sample where a different thiolate ligand, dodecanethiol (SC12), was used to prepare the clusters. This sample was shown to consist of both icosahedral and decahedral cores with the decahedral phase fraction being ∼14% (Jensen *et al.*, 2016 ▸). The resulting cluster-screen map is shown in Fig. 5 ▸. The *cluster-mining* methodology is stable, resulting in a cluster-screen map that is largely similar to the pure single-phase icosahedral SC6 sample shown in Fig. 4 ▸(*a*). It yields the 55-atom Mackay core as the best candidate cluster which is consistent with the expected majority phase, but the cluster-screen map also shows that the *R*_w_ trends for icosahedral and decahedral clusters have changed, with the two motifs reaching minima much closer to one another compared with the single-phase case. This behavior may be characteristic of nanoparticle mixtures. In the future we will explore extending *cluster-mining* to quantify minority phases in multi-phase samples.

## 4. Experimental methods   

Pd samples were prepared by the Murray group using methods described by Mazumder *et al.* (2012 ▸). Synthesis of Au_144_(SR)_60_ cluster samples was carried out in the Ackerson group following Qian & Jin (2011 ▸). Pd nanoparticle data were collected at the National Synchrotron Light Source II (beamline XPD, 28-ID-2) at Brookhaven National Laboratory and data for the two cluster samples, Au_144_(SC6)_60_ and Au_144_(SC12)_60_, were collected at the Advanced Photon Source (11-ID-B), Argonne National Laboratory. During both beamtimes, data were collected using the rapid acquisition PDF geometry (Chupas *et al.*, 2003 ▸) with large-area 2D detectors mounted behind nanopowder samples loaded in, or deposited on, polyimide capillaries and films. Pd nanoparticle samples were measured at 300 K with λ = 0.1846 Å and the two cluster samples were measured at 100 K with λ = 0.1430 Å.

*Fit2D* (Hammersley *et al.*, 1996 ▸; Hammersley, 2016 ▸) was used to calibrate experimental geometries and azimuthally integrate diffraction intensities to 1D diffraction patterns for all three samples. Standardized corrections were then made to the data to obtain the total scattering structure function, *F*(*Q*), which was then sine Fourier transformed to obtain the PDF, using *PDFgetX3* (Juhás *et al.*, 2013 ▸) within *xPDFsuite* (Yang *et al.*, 2015 ▸). The range of data used in the Fourier transform (*Q*_min_ to *Q*_max_, where 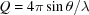 is the magnitude of the momentum transfer on scattering) was tuned per sample to give the best trade-off between statistical noise and real-space resolution, and also to truncate low-*Q* scattering unambiguously originating from organic species in the sample. For Pd nanoparticles, a range from 2.0 ≤ *Q* ≤ 26.0 Å^−1^ was used, and for the cluster samples ranges of 0.8 ≤ *Q* ≤ 27.0 Å^−1^ and 0.8 ≤ *Q* ≤ 26.0 Å^−1^ were used for Au_144_(SC6)_60_ and Au_144_(SC12)_60_, respectively.
